# The Urine Biomarker PUR-4 Is Positively Associated with the Amount of Gleason 4 in Human Prostate Cancers

**DOI:** 10.3390/life11111172

**Published:** 2021-11-03

**Authors:** Richard Y. Ball, Ryan Cardenas, Mark S. Winterbone, Marcelino Y. Hanna, Chris Parker, Rachel Hurst, Daniel S. Brewer, Lauren D’Sa, Rob Mills, Colin S. Cooper, Jeremy Clark

**Affiliations:** 1 Norfolk and Norwich University Hospitals NHS Foundation Trust, Norwich NR4 7UY, UK; richard.ball@nnuh.nhs.uk (R.Y.B.); lauren.dsa@doctors.org.uk (L.D.); robert.mills@nnuh.nhs.uk (R.M.); 2 Norwich Medical School, University of East Anglia, Norwich NR4 7TJ, UK; R.Cardenas@uea.ac.uk (R.C.); Mark.Winterbone@uea.ac.uk (M.S.W.); R.Hurst1@uea.ac.uk (R.H.); D.Brewer@uea.ac.uk (D.S.B.); Colin.Cooper@uea.ac.uk (C.S.C.); 3 Urology Department Castle Hill, Hull University Teaching Hospital, Castle Rd, Cottingham HU16 5JQ, UK; marcelino.hanna1@nhs.net; 4 Institute of Cancer Research, Sutton SM2 5NG, UK; Chris.Parker@icr.ac.uk; 5 Royal Marsden Hospital, Sutton SM2 5PT, UK; 6 Earlham Institute, Norwich NR4 7UZ, UK

**Keywords:** prostate, cancer, urine, PUR, PUR-4, Gleason pattern 4

## Abstract

The Prostate Urine Risk (PUR) biomarker is a four-group classifier for predicting outcome in patients prior to biopsy and for men on active surveillance. The four categories correspond to the probabilities of the presence of normal tissue (PUR-1), D’Amico low-risk (PUR-2), intermediate-risk (PUR-3), and high-risk (PUR-4) prostate cancer. In the current study we investigate how the PUR-4 status is linked to Gleason grade, prostate volume, and tumor volume as assessed from biopsy (*n* = 215) and prostatectomy (*n* = 9) samples. For biopsy data PUR-4 status alone was linked to Gleason Grade group (GG) (Spearman’s, *ρ* = 0.58, *p* < 0.001 trend). To assess the impact of tumor volume each GG was dichotomized into Small and Large volume cancers relative to median volume. For GG1 (Gleason Pattern 3 + 3) cancers volume had no impact on PUR-4 status. In contrast for GG2 (3 + 4) and GG3 (4 + 3) cancers PUR-4 levels increased in large volume cancers with statistical significance observed for GG2 (*p* = 0.005; Games-Howell). These data indicated that PUR-4 status is linked to the presence of Gleason Pattern 4. To test this observation tumor burden and Gleason Pattern were assessed in nine surgically removed and sectioned prostates allowing reconstruction of 3D maps. PUR-4 was not correlated with Gleason Pattern 3 amount, total tumor volume or prostate size. A strong correlation was observed between amount of Gleason Pattern 4 tumor and PUR-4 signature (*r* = 0.71, *p* = 0.034, Pearson’s). These observations shed light on the biological significance of the PUR biomarker and support its use as a non-invasive means of assessing the presence of clinically significant prostate cancer.

## 1. Introduction

Prostate cancer (PCa) diagnosis and prognosis are based on histopathological interpretation of biopsy cores to assess the grade and volume of cancer present as well as the relative proportions of the various Gleason patterns (GP) present. A number of studies have investigated the importance of amount of GP4 on the patient’s prognosis and survival. Stark et al. [1] indicated that Gleason Grade group (GG) 3 cancers which have a majority of GP4 cancer were associated with a three-fold higher rate of lethal outcome compared to GG2 cancers [1] which have a majority of GP3 cancer. Additional studies have highlighted that a binary cut-off for relative amount of GP4 cancer as used for defining GG2 and GG3, whilst useful, is not sufficient for complete prognostic discrimination. Specifically Choy et al. [2] reported that an increase in the percentage of tumor in radical prostatectomy (RPx) samples that is GP4 or greater is associated with an increase in biochemical recurrence (BCR)—ranging from 16% BCR in men where GP4 content was 1–20% to 68% BCR where GP4 was >70% [2]. The percentage of biopsy cores positive for tumor has also been found to link to prognosis [3] and is factored into the CAPRA (Cancer of the Prostate Risk Assessment) score [4]. There is further complexity in that tumor foci in up to 87% of RPx can contain multiple Gleason patterns [5,6,7].

Most tumors arise in the peripheral and transitional zones of the prostate, which are secretory in nature. Prostatic secretions carry tumor biomarkers into the urethra from where they can be harvested in urine. PCa-biomarkers in urine have shown utility in assessing prognosis. PCa-specific RNA transcripts such as *TMPRSS2:ERG* are detectable in urine from men with prostate cancer [8] and there has been much interest in development of a urine diagnostic test. The majority of such biomarker tests, including the PCA3 test (*PCA3* and *KLK3*), MiPS (serum PSA plus urine *TMPRSS2:ERG* and *PCA3*) and ExosomeDX Prostate Intelliscore (*ERG*, *PCA3*, *SPDEF*), rely on detecting transcripts from a very small number of genes which may not be expressed in every cancer [9,10,11]. In contrast we have developed the Prostate Urine Risk (PUR) signatures using a NanoString panel of 36 gene probes [12]. PUR detected PCa in urine samples that were negative for *PCA3* and/or *TMPRSS2:ERG*. The PUR signatures were designed to correspond to patients in four clinical groups: those with no evidence of cancer (NEC, PUR-1), and three D’Amico categories of PCa: low-(PUR-2), intermediate-(PUR-3) and high-risk PCa (PUR-4). The PUR-4 signature could detect intermediate and high-risk disease (AUC = 0.77, 95% confidence interval [CI] 0.70–0.84) and was able to predict disease progression in men on an active surveillance monitoring regime up to 5 years after a single urine sample was collected (HR 8.23, 95% CI 3.26–20.81; *p* < 0.001). To understand how PUR-4 related to the structure of the cancerous prostate we now examine its relationship to the amounts and grade of tumor in biopsy-sampled prostates and in a series of radical prostatectomy specimens.

## 2. Materials and Methods

### 2.1. Urine RNA Extraction and PUR Signatures

All urine samples (≤30 mL) were collected from 295 men who attended urology clinics at the Norfolk and Norwich University Hospitals NHS Foundation Trust (NNUH) UK (*n* = 214) and at the Royal Marsden Hospital (RMH) UK (*n* = 81) between September 2012 to September 2014 [12]. Ethical approval for this study was gained from the Health Research Authority and the NRES Committee East of England in 2012, REC reference: 12/EE/0058, IRAS project ID: 196199. Informed consent was obtained from all subjects involved in the study. All urine samples (≤30 mL) were collected pre-biopsy. Urine cell-free RNA (cfRNA) was extracted and the PUR signatures were generated from NanoString (NanoString Technologies Inc, Seattle, WA, USA) expression analysis as per Connell et al. (2019) [12].

### 2.2. Biopsy Tissue Analyses

Full biopsy data required for tumor volume analysis (prostate volume, total number of cores taken and number of cores positive for PCa) were available for 215/295 patients (Table 1). The majority of the biopsies were trans-rectal ultrasound guided (TRUS) with a range of 2–23 cores taken per prostate (median 9, mean 9). Biopsy data were recorded as part of a standard biopsy assessment for PCa diagnosis at the NNUH and the RMH. Ultrasound prostate volumes ranged from 5.5–353.9 g (mean 53.9, SD 41.9). For clinical characteristics of Gleason grade groups divided into small and large tumors see Supplementary Table S1.

### 2.3. Radical Prostatectomy Analyses

**Figure 1:**
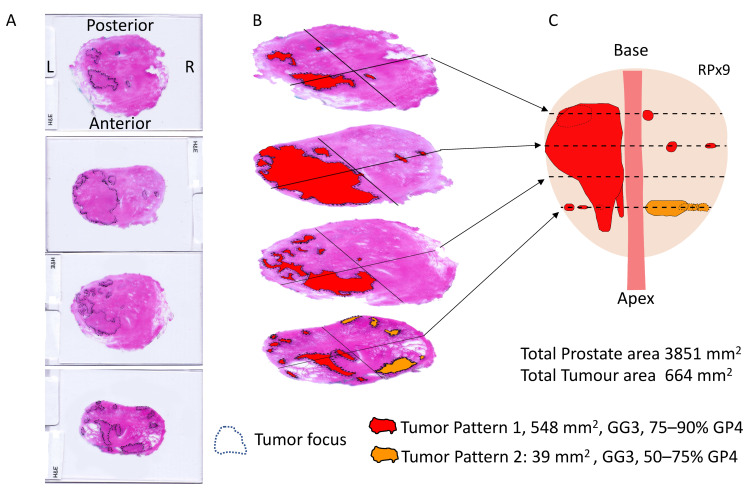
Assessment of prostatectomy RPx9. (**A**) RPx9 was cut into 4 sections, for which H&E slides were prepared and areas of tumor marked up. (**B**) the sections are arranged for viewing from base to apex. The PCa areas have been colored to correspond to the Gleason Grade Group (GG) and % GP4 found: red indicates GG3 with 75–90% GP4, orange indicates GG3 with 50–75% GP4 (see Figure 2). (**C**) the information in ‘B’ was used to create a 3D projection of what the prostate and tumor may have looked like. Dotted tumor indicates a tumor focus behind a more anterior tumor.

**Figure 2:**
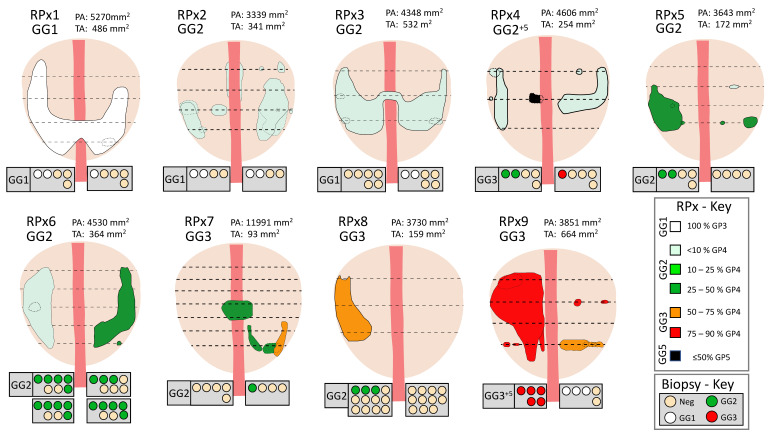
Image projections of tumor content for 9 radical prostatectomies (RPx1–9). Images were constructed for each prostate as shown in Figure 1. The different colors correspond to the % GP4 detected (RPx-Key). PA: prostate area summed for all slices. TA: Tumor Area. ‘+5’ indicates a tertiary region of GP5 tumor. Dotted tumor indicates a tumor focus behind a more anterior tumor. Horizontal dotted lines indicate approximate positions of H&E sections examined. The grey boxes underneath each 3D construct contain the biopsy data taken prior to prostatectomy, including the highest GG found on biopsy. Each circle represents a core taken, data is divided up into left and right cores and also into anterior (upper boxes) and posterior (lower boxes) for one prostate (RPx6).

Nine men had a radical prostatectomy (RPx) within 4 months of their biopsy, and these were analyzed in detail. The RPx were fixed, embedded, sectioned at ~5mm intervals and megablock slides were stained with hematoxylin and eosin (H&E) as part of the standard histopathological reporting process for the NNUH. The H&E stained megablock sections were re-examined for this study by two NNUH histopathologists (RYB, LDS) and marked to indicate all areas of tumor. Each tumor focus was given a Gleason grade (GG) and an estimate for the percentage of Gleason Pattern (GP) 3 and 4 present (see main text and Figure 1). GG2 and GG3 tumor foci were then divided into 6 groups, depending on the percent of Gleason 4 present (see Figure 1 and Figure 2). The tumor-marked H&E-stained slides were scanned on a flatbed scanner at 300 dpi, and Fiji software (Image J version 1.42q, https://imagej.net/software/fiji/) was used to analyze the images and measure the areas of prostate and of each focus of cancer. Prior to analysis of each tissue section Fiji was calibrated using the known width of the H&E slide in the scanned image. Total areas of GP3 and GP4 tumor in each prostate were calculated by multiplying area of each tumor foci by the percentage of GP3 or GP4 present.

### 2.4. Statistical Analyses

R (v4.0.3) was utilized to perform all statistical analyses. Prior to test selection data were checked for appropriateness for normality and variance using QQ-plots and flinger/shapiro tests, respectively, using the base R package. The Rstatix package was used to perform Kruskal-Wallis rank sum test and Games Howell post-hoc test. All correlation analyses were performed using base R and used Cohen’s effect sizes [13]. Plots were developed using ‘ggplot’ in combination with multiple package extensions including ‘ggsci’, ‘ggrepel’ and ‘ggpubr’. The collection of ‘tidyverse’ packages were utilized for data manipulation. Default options were opted for unless otherwise stated.

## 3. Results

### 3.1. Analyses of Prostate Needle Biopsy Tissue

**Figure 3:**
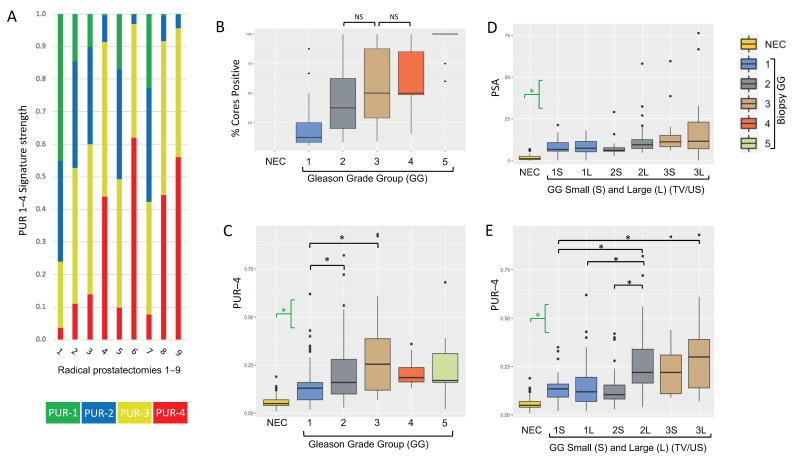
PUR status in biopsy samples. (**A**) Distribution of the four PUR signatures from nine patients that were subsequently treated by radical prostatectomy. See Figure 1 and Figure 2 for the cancer maps corresponding to these cases. PUR-1 (green), PUR-2 (blue), PUR-3 (yellow), PUR-4 (red). The sum of PUR-1-4 in each sample is ‘1’. (**B**–**D**) Data were analyzed using a non-parametric Kruskal-Wallis analysis of variance followed by Games-Howell post-hoc analysis. (**B**) % Biopsy cores positive also referred to as tumor volume (TV) was calculated as PCa-positive cores/total cores taken. The clinical categories are ‘NEC’: No Evidence of Cancer (*n* = 62) and Biopsy Gleason Grade (GG) groups GG1 (*n* = 93), GG2 (*n* = 61), GG3 (*n* = 38), GG4 (*n* = 12), GG5 (*n* = 11). ‘NS’ indicates ‘Not Significant’ difference in pair-wise analysis of the groups, all other pairwise comparisons were significant (*p* < 0.05). (**C**) PUR-4 signature for samples in Figure 3B. * indicates a significant difference between groups. Green brackets indicate that a significant difference was found between NEC patient samples and each of the GG patient groups. (**D**) Serum PSA in GG 1, 2, 3 samples dichotomized based on tumor volume/ultrasound prostate volume (TV/US) into small (S) and large (L) groups, only pairwise comparisons with NEC were significant. No significant differences between GG (S) and (L) groups were found. (**E**) PUR-4 signal for samples dichotomized as ‘D’ above.

PUR signatures (PUR-1 to PUR-4) were generated as published in Connell et al. [12] from urine cell-free-RNA NanoString expression data (36 NanoString gene probes) collected from patients with a suspicion of prostate cancer (Movember cohort, *n* = 535). PUR signatures represent the probability of membership of each of the following clinical groups: PUR-1, NEC (No clinical Evidence of Cancer, PSA normal for age); and PUR-2 to 4, respectively the three D’Amico risk categories low-(PUR-2), intermediate-(PUR-3) and high-risk (PUR-4) [12]. Each sample is represented by a readout in all four PUR signatures with the sum adding up to ‘1’ (see Figure 3A for examples).

The ‘Biopsy cohort’ consisted of men with PCa detected on biopsy in the Movember cohort for whom full biopsy data were available: number of cores positive; Gleason score; pre-biopsy PSA; and prostate ultrasound volume, *n* = 215. Additionally, 62 men designated as NEC from the Movember cohort were used in comparisons of PUR values. PUR-4 correlated with increasing D’Amico risk group (Supplementary Figure S1), and PUR-4 was significantly different in pairwise comparisons between NEC, D’Amico low, and intermediate-risk groups. PSA also correlated with increasing D’Amico risk group and was significantly different in pairwise comparisons between all clinical categories (Figure S1). Comparison of PUR-4 to DRE data was not performed due to unreliability of data (see discussion). As expected, the percentage of cores positive for prostate cancer correlated with Gleason Grade group (GG) (Spearman’s coefficient of rank correlation *ρ* = 0.63, *p* = 1.77 × 10^−25^, Figure 3B). PUR-4 signature values showed a significant positive correlation with increasing GG (Spearman’s correlation *ρ* = 0.58, *p* = 3.5 × 10^−24^, Figure 3C). When intercomparing the different clinical groups, PUR-4 was significantly different between NEC and each individual GG (all *p* < 0.00035); GG1 cancers were significantly different to both GG2 (Games-Howell post-hoc test, *p* = 0.003) and GG3 tumors (*p* = 0.036). There was no significant difference in tumor volume or PUR-4 between GG2 and GG3 tumors (both *p* > 0.05; Games-Howell post-hoc test).

We next examined the relationships between PUR-4 value and tumor volume. To achieve this the results for each GG were divided into large (L) and small (S) volume cancers relative to the median tumor volume. Tumor volume was calculated as the number of PCa-positive cores/total number of cores taken, with individual values corrected for their patient’s prostate volume determined by ultrasound (US). GG4 (*n* = 12) and GG5 groups (*n* = 11) were not subdivided due to the small numbers of samples. There were no significant differences in PSA between (S) and (L) groups for GG1, GG2 or GG3 (Figure 3D, Games-Howell post-hoc test, all *p* > 0.05). There was no difference in PUR-4 values comparing GG1(S) and GG1(L) (Games-Howell post-hoc test, *p* = 0.98, Figure 3E). Since GG1 cancers contain only Gleason Pattern 3 cancer this observation showed that the volume of Gleason Pattern 3 cancer present had no impact on PUR-4 values. PUR-4 for GG2 (L) was significantly greater than for GG2 (S) (*p* = 0.005; Games-Howell post-hoc test), while PUR-4 values for GG3 (L) were greater than for GG3 (S) but did not reach significance (Figure 3E). Since GG2 and GG3 contain both Gleason Pattern 3 and 4 cancer these observations suggest that Gleason Pattern 4 cancer may be contributing to PUR4 status.

### 3.2. Prostatectomy Analyses

Nine radical prostatectomy specimens were chosen for histopathological re-examination; three GG1, four GG2, and two GG3 based on pre-surgical histopathological assessment of biopsies. H&E analysis was carried out on all individual sections from each RPx as shown in Figure 1 allowing construction of 3D cancer maps (Figure 2). Each section was examined independently by 2 histopathologists (RYB and LD) and areas of cancers were marked depending on the estimated percentage of GP4 present: (i) <10% GP4; (ii) 10–25% GP4; (iii) 26–50% GP4; (iv) 51–75% GP4; (v) 76–90% GP4; and (vi) >90% GP4 (Figure 1 and Figure 2). For each focus of tumor the range midpoint was multiplied by the focus area to provide an estimated amount of GP3 and GP4. These values were summed for each prostate (Table 2, biopsy data for RPx1-9 are presented in Supplementary Table S2.

**Figure 4:**
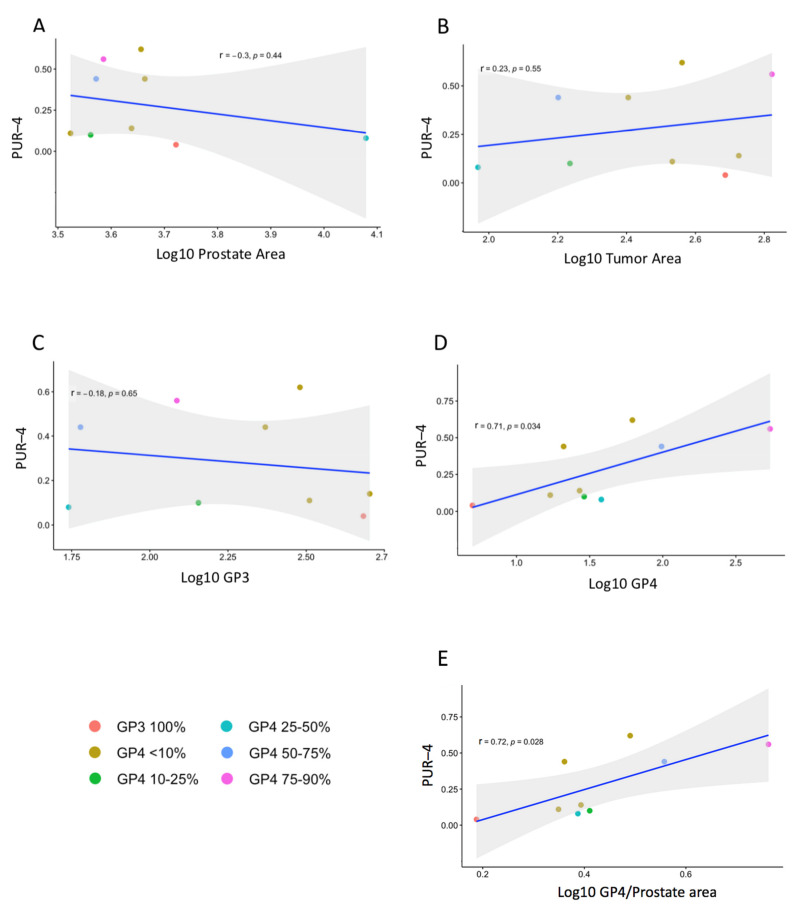
Correlation of PUR-4 with prostate parameters. Measurements were taken from the 9 whole mount prostate H&E sections shown in Figure 2. Each sample is colored by the Gleason score of the predominant tumor area (see key). No correlation was found between PUR-4 and (**A**) prostate area of the H&E sections, (**B**) tumor area, (**C**) amount of Gleason pattern 3. (**D**) PUR-4 correlated with amount of Gleason pattern 4 cancer both before, and (**E**) after adjustment for prostate area (see body text for details). ‘*r*’ is the Pearson’s correlation coefficient, an ‘*r*’ > 0.7 indicates a strong correlation, ‘*p*’ = probability that the correlation has not arisen by chance.

No significant correlation was observed between PUR-4 score and PSA (Figure S1), or the size of the prostate as measured by ultrasound or H&E tissue area (Figure 4), (Pearson’s correlation, *r* = −0.33 *p* = 0.4; *r* = −0.47 *p* = 0.2; *r* = –0.3 *p* > 0.4 respectively). No significant associations were identified between PUR-4 scores and the total area of tumor or the area of GP3 tumor with or without adjustment for prostate size (Pearson’s correlation all *p* ≥ 0.48) (Figure 4). The amount of GP4 tumor was found to have a strong significant positive correlation with PUR-4 score (*r* = 0.71, *p* = 0.035; Pearson’s correlation; Figure 4D), and similar strong associations were found after adjustment for total prostate area on H&E slides (*r* = 0.72, *p* = 0.028; Figure 4E) or for ultrasound prostate volume (*r* = 0.73, *p* = 0.024).

## 4. Discussion

When estimating Gleason Grade Evans et al. [14] reported that only 55% of cases were concordant between biopsy and radical prostatectomy, while Yang et al. [15] and Epstein et al. [16] reported upgrading in 30–36% of RPxs. Comparably we found disease upgrading in 55% (5 of 9) prostatectomy specimens (Figure 2). Two out of three RPx reported as GG1 on biopsy had GP4 in their RPxs, 2/4 biopsy GG2 were upgraded to GG3, and GP5 was found in one prostate (RPx4). As the interval between biopsy and prostatectomy was ≤4 months for all samples, these Gleason differences were inferred to be due to biopsy sampling issues. Despite potential limitations of the tumor volume assessment, our results support the hypothesis that there is a relationship between increasing amounts of GP4 PCa and increasing PUR-4 signal in GG2 and GG3 tumors both in biopsy specimens and for prostatectomies. The importance of detecting and estimating the amount of Gleason pattern 4 tumor is highly relevant to risk stratification and selecting the appropriate treatment pathway for a patient [17,18,19].

No difference in PUR-4 signals were observed between GG2 (S) and the GG1 (S) and GG1 (L) subgroups (*p* > 0.05). We have previously shown that a PUR-4 signature of <0.174 identified a subgroup of men whose disease did not progress to require treatment intervention up to 5 years later (HR 8.23, 95% CI 3.26–20.81; *p* < 0.001) [12]. Significantly, 80% of the GG2 (S) tumors were below the 0.174 PUR-4 cutoff, and the vast majority of these (83%) had <30% PCa-positive cores (average 21%, median 19%), a figure comparable to that found in the GG1 cancers (94% <30% cores positive, median 13%, average 15%). These results suggest that PUR-4 may be identifying a group of low tumor-volume GG2 men who could benefit from an active surveillance monitoring regime.

One limitation of this study is that we could not interrogate an increase in PUR-4 in patients with GG4 and GG5 disease due to the small sample numbers involved. Anecdotally the PUR-4 signature for RPx4 which was found to have an area of GP4+5 on RPx was much higher than expected for the volume of GP4 calculated in this largely GG2 < 10% GP4 cancer, which may imply that PUR-4 can respond to disease that is becoming more aggressive.

PUR-4 increases with D’Amico risk group, and D’Amico categorization includes data from Gleason, PSA and DRE. However, while PSA increased with D’Amico risk group, there were no differences in PSA between the GG (S) and GG (L) groups. In addition, there was no correlation between PSA and PUR-4 for the 9 RPx. When taken together these results indicate that PUR-4 does not appear to mirror serum PSA. DRE information was not considered reliable data for this cohort, DRE data is considered to be subjective, for example Gosselaar et al. [20] found that suspicion of cancer on DRE by 6 clinicians varied from 4–28%, while Ankerst [21] found that 70% of abnormal DREs were DRE-normal the following year. A metanalysis by Naji et al. [22] of 8217 studies led them to the conclusion that there was no evidence for the efficacy of using a DRE to detect prostate cancer [22]. The percentage of cores positive was considered to be a more solid assessment of extent of tumor, and as approximately equal numbers of cores were taken on each side of the prostate, a figure of >50% cancer positive would indicate disease on both sides of the prostate.

We propose that the PUR urine test could help in the diagnostic pathway for patients who are worried about their risk of prostate cancer and those with suspected prostate cancer. mpMRI has aided the diagnosis of significant disease, but negative aspects of mpMRI are that it can miss significant cancers (GP ≥ 4) [23], identify insignificant cancers (GG1) [24], and has been reported to have a high false-positive rate of around 50% [23], with problems of interoperator inconsistencies [25]. We believe that the PUR test could add to the information from multiparametric magnetic resonance imaging (mpMRI) and aid decision making on whether a biopsy is necessary.

## 5. Conclusions

A strong association was observed between an increasing amount of Gleason pattern 4 cancer and increasing PUR-4 signal. These data suggest that the PUR-4 signature could provide useful additional information in determining the amount of clinically significant tumor within a prostate and thereby help guide the patient treatment pathway with essential information for triage, improved management and prognostic utility.

## Figures and Tables

**Table 1:** Clinical characteristics and PUR scores for biopsy cases. All data presented as: ‘Median (IQR)’.

	NEC	GG1	GG2	GG3	GG4	GG5
Total (%)	62 (22.4%)	93 (33.6%)	61 (22.0%)	38 (13.7%)	12 (4.3%)	11 (4.0%)
Age in years	66 (17)	67 (6.5)	68 (9)	73 (8.25)	71 (12.25)	69 (8.5)
PSA ng/mL	1.2 (2)	7.3 (5.5)	7.8 (4.5)	11.35 (11.03)	13.45 (12.7)	18.7 (45.95)
Prostate volume US	NA	23 (7.5)	42.43 (24.7)	49.03 (28.4)	50.39 (22.9)	55.93 (24.97)
PSAD	NA	0.292 (0.31)	0.179 (0.12)	0.256 (0.22)	0.287 (0.38)	0.31 (0.56)
Biopsy Cores taken	NA	11 (4)	9 (2)	8 (6)	5.5 (5.25)	4 (6.5)
Biopsy Cores positive	NA	1 (1)	3 (3)	3.5 (2.75)	3.5 (3.5)	4 (5)
% biopsy cores positive	NA	12.5 (16.67)	37.5 (42.5)	50 (58.57)	50 (36.39)	100 (0)
% biopsy cores positive/US	NA	0.003 (0.004)	0.001 (0.001)	0.001 (0.001)	0.001 (0.001)	0.001 (0.002)
PUR-1	0.358 (0.206)	0.119 (0.165)	0.078 (0.145)	0.03 (0.119)	0.059 (0.04)	0.07 (0.059)
PUR-2	0.321 (0.036)	0.302 (0.078)	0.275 (0.151)	0.187 (0.217)	0.248 (0.07)	0.201 (0.124)
PUR-3	0.256 (0.129)	0.442 (0.15)	0.452 (0.116)	0.475 (0.119)	0.506 (0.032)	0.489 (0.073)
PUR-4	0.049 (0.036)	0.126 (0.09)	0.162 (0.18)	0.255 (0.269)	0.188 (0.075)	0.171 (0.146)

Abbreviations: IQR, interquartile range; GG, Gleason Grade; NEC, No Evidence of Cancer; US, Ultrasound; Age and PSA at time of urine collection; PSA, prostate specific antigen; PSAD, PSA density (PSA/US prostate volume). PUR-1–4, Prostate urine risk signatures 1–4, NA, Not applicable.

**Table 2:** Clinical characteristics for the 9 radical prostatectomy (RPx) cases examined.

	GG	Majority GG, %GP4	Prostate Area (mm^2^)	Tumor Area (mm^2^)	GP3 Area	GP4 Area	Age	PSA	PUR-4
RPx 1	GG1	GG1^+4^	5269.9	486.4	481.6	4.8	54	5.5	0.04
RPx 2	GG2	GG2, <10% GP4	3339.0	341.2	324.1	17.1	64	15	0.11
RPx 3	GG2	GG2, <10% GP4	4347.6	531.8	505.2	26.6	52	5.8	0.14
RPx 4	GG2^+5^	GG2, <10% GP4	4606.0	254.5	233.9	20.5	70	8.4	0.44
RPx 5	GG2	GG2, 10–25% GP4	3643.2	172.1	143.3	28.8	66	5.2	0.10
RPx 6	GG2	GG2, <10% GP4	4529.7	364.0	302.0	62.0	68	6.7	0.62
RPx 7	GG3	GG2, 25–50% GP4	11,991.1	92.9	54.8	38.0	67	10.3	0.08
RPx 8	GG3	GG3, 50–75% GP4	3729.6	158.7	60.3	98.4	65	7.4	0.44
RPx 9	GG3	GG3, 75–90% GP4	3851.4	664.4	122.2	542.2	75	2.9	0.56

GG = Gleason Grade group; Majority, data for most common GG found in each prostate; GP3, GP4, GP5, Gleason Pattern 3, 4, 5 respectively; Age, age at recruitment; US, Ultrasound; PCa, prostate cancer; All areas measured from scans of H&E slides (see methods); for GP3 and GP4 amount calculation see results; GG2^+5^ indicates GG2 cancer with a tertiary GP5 (<5% of all PCa), similarly GG1^+4^ indicates a GG1 cancer with tertiary GP4; PUR-4, Prostate urine risk signatures 4.

## Data Availability

Data is contained within the article or supplementary material.

## Supplementary Materials

The following are available online at https://www.mdpi.com/article/10.3390/life11111172/s1, Figure S1: PSA and PUR-4 data in biopsy patients categorized on D’Amico and PSA in the nine RPx men, Table S1: Clinical characteristics and PUR scores for biopsy cases categorized by Gleason Grade and subcategorized by % biopsy cores positive, Table S2: Combined RPx and Biopsy clinical characteristics for the 9 radical prostatectomy (RPx) cases examined.

## References

[1] Stark J.R., Perner S., Stampfer M.J., Sinnott J.A., Finn S., Eisenstein A.S., Ma J., Fiorentino M., Kurth T., Loda M. (2009). Gleason score and lethal prostate cancer: Does 3 + 4 = 4 + 3?. J. Clin. Oncol..

[2] Choy B., Pearce S.M., Anderson B.B., Shalhav A.L., Zagaja G., Eggener S.E., Paner G.P. (2016). Prognostic Significance of Percentage and Architectural Types of Contemporary Gleason Pattern 4 Prostate Cancer in Radical Prostatectomy. Am. J. Surg. Pathol..

[3] Spalding A.C., Daignault S., Sandler H.M., Shah R.B., Pan C.C., Ray M.E. (2007). Percent positive biopsy cores as a prognostic factor for prostate cancer treated with external beam radiation. Urology.

[4] Cooperberg M.R., Freedland S.J., Pasta D.J., Elkin E.P., Presti J.C., Amling C.L., Terris M.K., Aronson W.J., Kane C.J., Carroll P.R. (2006). Multiinstitutional validation of the UCSF cancer of the prostate risk assessment for prediction of recurrence after radical prostatectomy. Cancer.

[5] Arora R., Koch M.O., Eble J.N., Ulbright T.M., Li L., Cheng L. (2004). Heterogeneity of Gleason grade in multifocal adenocarcinoma of the prostate. Cancer.

[6] Le J.D., Tan N., Shkolyar E., Lu D.Y., Kwan L., Marks L.S., Huang J., Margolis D.J.A., Raman S.S., Reiter R.E. (2015). Multifocality and prostate cancer detection by multiparametric magnetic resonance imaging: Correlation with whole-mount histopathology. Eur. Urol..

[7] Cooper C.S., Eeles R., Wedge D.C., Van Loo P., Gundem G., Alexandrov L.B., Kremeyer B., Butler A., Lynch A.G., Camacho N. (2015). Analysis of the genetic phylogeny of multifocal prostate cancer identifies multiple independent clonal expansions in neoplastic and morphologically normal prostate tissue. Nat. Genet..

[8] Nguyen P.-N., Violette P., Chan S., Tanguay S., Kassouf W., Aprikian A., Chen J.Z. (2010). A Panel of TMPRSS2:ERG Fusion Transcript Markers for Urine-Based Prostate Cancer Detection with High Specificity and Sensitivity. Eur. Urol..

[9] Groskopf J., Aubin S.M.J., Deras I.L., Blase A., Bodrug S., Clark C., Brentano S., Mathis J., Pham J., Meyer T. (2006). APTIMA PCA3 molecular urine test: Development of a method to aid in the diagnosis of prostate cancer. Clin. Chem..

[10] Tomlins S.A., Day J.R., Lonigro R.J., Hovelson D.H., Siddiqui J., Kunju L.P., Dunn R.L., Meyer S., Hodge P., Groskopf J. (2016). Urine TMPRSS2:ERG Plus PCA3 for Individualized Prostate Cancer Risk Assessment. Eur. Urol..

[11] McKiernan J., Donovan M.J., Margolis E., Partin A., Carter B., Brown G., Torkler P., Noerholm M., Skog J., Shore N. (2018). A Prospective Adaptive Utility Trial to Validate Performance of a Novel Urine Exosome Gene Expression Assay to Predict High-grade Prostate Cancer in Patients with Prostate-specific Antigen 2–10 ng/mL at Initial Biopsy. Eur. Urol..

[12] Connell S.P., Hanna M., McCarthy F., Hurst R., Webb M., Curley H., Walker H., Mills R., Ball R.Y., Sanda M.G. (2019). A Four-Group Urine Risk Classifier for Predicting Outcome in Prostate Cancer Patients. BJU Int..

[13] Cohen J. (2013). Statistical Power Analysis for the Behavioral Sciences.

[14] Evans S.M., Bandarage V.P., Kronborg C., Earnest A., Millar J., Clouston D. (2016). Gleason group concordance between biopsy and radical prostatectomy specimens: A cohort study from Prostate Cancer Outcome Registry-Victoria. Prostate Int..

[15] Yang D.D., Mahal B.A., Muralidhar V., Nezolosky M.D., Vastola M.E., Labe S.A., Boldbaatar N., King M.T., Martin N.E., Orio P.F. (2019). Risk of Upgrading and Upstaging Among 10,000 Patients with Gleason 3 + 4 Favorable Intermediate-risk Prostate Cancer. Eur. Urol. Focus.

[16] Epstein J.I., Feng Z., Trock B.J., Pierorazio P.M. (2012). Upgrading and Downgrading of Prostate Cancer from Biopsy to Radical Prostatectomy: Incidence and Predictive Factors Using the Modified Gleason Grading System and Factoring in Tertiary Grades. Eur. Urol..

[17] Kane C.J., Eggener S.E., Shindel A.W., Andriole G.L. (2017). Variability in Outcomes for Patients with Intermediate-risk Prostate Cancer (Gleason Score 7, International Society of Urological Pathology Gleason Group 2–3) and Implications for Risk Stratification: A Systematic Review. Eur. Urol. Focus.

[18] Sanda M.G., Cadeddu J.A., Kirkby E., Chen R.C., Crispino T., Fontanarosa J., Freedland S.J., Greene K., Klotz L.H., Makarov D.V. (2017). Clinically Localized Prostate Cancer: AUA/ASTRO/SUO Guideline. Part I: Risk Stratification, Shared Decision Making, and Care Options. J. Urol..

[19] Zumsteg Z.S., Zelefsky M.J. (2012). Short-term androgen deprivation therapy for patients with intermediate-risk prostate cancer undergoing dose-escalated radiotherapy: The standard of care?. Lancet Oncol..

[20] Gosselaar C., Kranse R., Roobol M.J., Roemeling S., Schröder F.H. (2008). The interobserver variability of digital rectal examination in a large randomized trial for the screening of prostate cancer. Prostate.

[21] Ankerst D.P., Miyamoto R., Nair P.V., Pollock B.H., Thompson I.M., Parekh D.J. (2009). Yearly prostate specific antigen and digital rectal examination fluctuations in a screened population. J. Urol..

[22] Naji L., Randhawa H., Sohani Z., Dennis B., Lautenbach D., Kavanagh O., Bawor M., Banfield L., Profetto J. (2018). Digital Rectal Examination for Prostate Cancer Screening in Primary Care: A Systematic Review and Meta-Analysis. Ann. Fam. Med..

[23] Ahmed H.U., El-Shater Bosaily A., Brown L.C., Gabe R., Kaplan R., Parmar M.K., Collaco-Moraes Y., Ward K., Hindley R.G., Freeman A. (2017). Diagnostic accuracy of multi-parametric MRI and TRUS biopsy in prostate cancer (PROMIS): A paired validating confirmatory study. Lancet.

[24] Eldred-Evans D., Burak P., Connor M.J., Day E., Evans M., Fiorentino F., Gammon M., Hosking-Jervis F., Klimowska-Nassar N., McGuire W. (2021). Population-Based Prostate Cancer Screening with Magnetic Resonance Imaging or Ultrasonography. JAMA Oncol..

[25] Walz J. (2018). The “PROMIS” of Magnetic Resonance Imaging Cost Effectiveness in Prostate Cancer Diagnosis?. Eur. Urol..

