# Supplementary material for: The Urine Biomarker PUR-4 Is Positively Associated with the Amount of Gleason 4 in Human Prostate Cancers
Source: Life (Basel). 2021 Nov 3;11(11):1172. doi: 10.3390/life11111172 (PMC8622091; doi:10.3390/life11111172)
Supplement: Supplementary file 1 [file life-11-01172-s001.zip › life-1424157-supplementary.pdf]

life

MDPI

Supplementary materials

The Urine Biomarker PUR-4 is Positively Associated with the Amount of Gleason 4 in Human Prostate Cancers.

Richard Y. Ball, Ryan Cardenas, Mark S. Winterbone, Marcelino Y. Hanna, Chris Parker, Rachel Hurst, Daniel S. Brewer, Lauren D'Sa, Rob Mills, Colin S. Cooper and Jeremy Clark

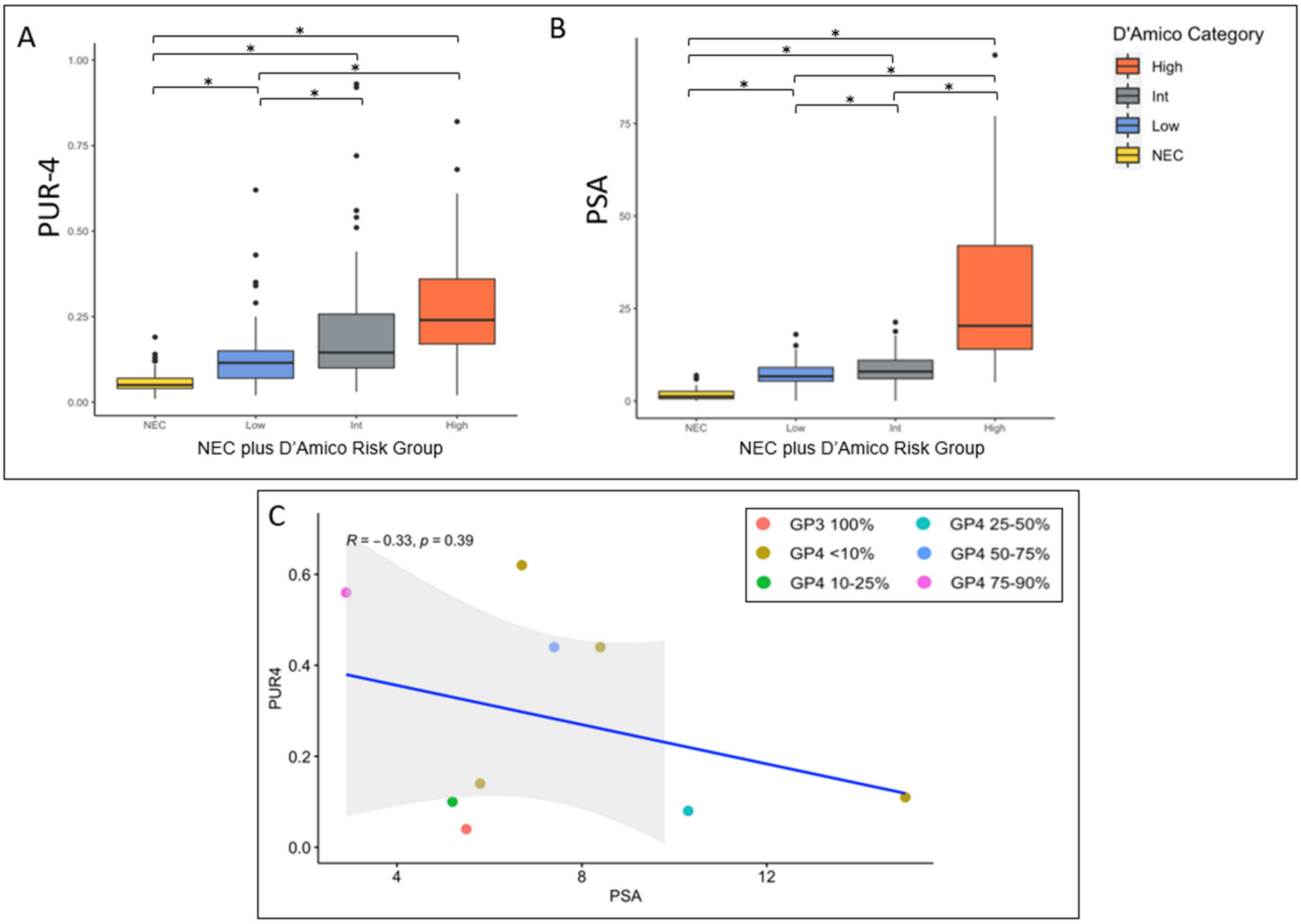

Figure S1. PSA and PUR-4 data in biopsy patients categorized on D'Amico and PSA in the nine RPx men**.** For 'a' and 'b' Data were analyzed using a non-parametric Kruskal-Wallis analysis of variance followed by Games-Howell post-hoc analysis. A) PUR-4 in men categorized as No Evidence of prostate Cancer (NEC) and D'Amico risk groups low, intermediate and high-risk. PUR-4 correlated with increasing D'Amico risk group (Pearson's correlation r=0.62, p=3.2e^{-30}). '*' indicates a significant difference ($p<0.05$). B) PSA correlated with increasing D'Amico risk group (Pearson's correlation r=0.73, p=9.8e^{-47}). C) PUR-4 and PSA for the nine prostatectomy cases, no correlation was found (rho = -0.33, p=0.39).

Table S1. Clinical characteristics and PUR scores for biopsy cases categorized by Gleason Grade and subcategorized by % biopsy cores positive. All data presented as: 'Median (IQR)', Abbreviations: IQR, interquartile range; GG, Gleason Grade; NEC, No Evidence of Cancer; US, Ultrasound; Age and PSA at time of urine collection; PSA, prostate specific antigen; PSAD, PSA density (PSA/US prostate volume). PUR-1-4, Prostate urine risk signatures 1-4, NA, Not applicable.

	**NEC**	**GG1**	**GG2**	**GG3**	**GG4**	**GG5**	**GG1(S)**	**GG1(L)**	**GG2(S)**	**GG2(L)**	**GG3(S)**	**GG3(L)**
Total (%)	62 (22.4%)	93 (33.6%)	61 (22.0%)	38 (13.7%)	12 (4.3%)	11 (4.0%)	46 (16.6%)	47 (17%)	30 (10.8%)	31 (11.2%)	19 (6.9%)	19 (6.9%)
Age in years,	66 (17)	67 (6.5)	68 (9)	73 (8.25)	71 (12.25)	69 (8.5)	67 (8)	69.5 (8.25)	67.5 (9.5)	69 (7)	75 (7)	70 (10)
PSA ng/ml	1.2 (2)	7.3 (5.5)	7.8 (4.5)	11.35 (11.03)	13.45 (12.68)	18.7 (45.95)	6.75 (5.05)	7.5 (6.2)	6.2 (2.15)	9.5 (5.25)	11.2 (6.75)	11.6 (15.9)
Prostate volume US	NA	23 (7.5)	42.43 (24.73)	49.03 (28.42)	50.39 (22.89)	55.93 (24.97)	25.08 (32.45)	22 (5.25)	55.29 (33.62)	37.95 (20.96)	59 (43.28)	35.94 (18.03)
PSAD	NA	0.292 (0.315)	0.179 (0.122)	0.256 (0.227)	0.287 (0.385)	0.31 (0.565)	0.248 (0.272)	0.417 (0.392)	0.123 (0.055)	0.24 (0.309)	0.19 (0.098)	0.323 (0.454)
biopsy Cores taken	NA	11 (4)	9 (2)	8 (6)	5.5 (5.25)	4 (6.5)	12 (2)	9 (4)	10 (2.75)	8 (2)	10 (3)	6 (4)
Biopsy Cores positive	NA	1 (1)	3 (3)	3.5 (2.75)	3.5 (3.5)	4 (5)	1 (0)	2 (1.5)	2 (2)	5 (2.5)	2 (2)	4 (2.5)
% biopsy cores positive	NA	12.5 (16.67)	37.5 (42.5)	50 (58.57)	50 (36.39)	100 (0)	8.333 (4.17)	18.182 (16.67)	20 (14.21)	62.5 (25)	28.571 (31.79)	80 (35.42)
% biopsy cores positive/US	NA	0.003 (0.004)	0.001 (0.001)	0.001 (0.001)	0.001 (0.001)	0.001 (0.002)	0.003 (0.004)	0.003 (0.004)	0.001 (0)	0.001 (0.001)	0.001 (0)	0.001 (0.002)
PUR-1	0.358 (0.206)	0.119 (0.165)	0.078 (0.145)	0.03 (0.119)	0.059 (0.04)	0.07 (0.059)	0.112 (0.104)	0.129 (0.202)	0.152 (0.135)	0.041 (0.061)	0.042 (0.126)	0.019 (0.094)
PUR-2	0.321 (0.036)	0.302 (0.078)	0.275 (0.151)	0.187 (0.217)	0.248 (0.07)	0.201 (0.124)	0.302 (0.06)	0.306 (0.099)	0.315 (0.059)	0.214 (0.143)	0.217 (0.178)	0.152 (0.197)
PUR-3	0.256 (0.129)	0.442 (0.15)	0.452 (0.116)	0.475 (0.119)	0.506 (0.032)	0.489 (0.073)	0.45 (0.102)	0.417 (0.166)	0.411 (0.127)	0.491 (0.098)	0.473 (0.123)	0.483 (0.091)
PUR-4	0.049 (0.036)	0.126 (0.09)	0.162 (0.18)	0.255 (0.269)	0.188 (0.075)	0.171 (0.146)	0.131 (0.068)	0.119 (0.126)	0.107 (0.076)	0.223 (0.174)	0.219 (0.195)	0.302 (0.251)

Table S2. Combined RPx and Biopsy clinical characteristics for the 9 radical prostatectomy (RPx) cases examined. GG = Gleason Grade group; Majority, data for most common GG found in each prostate; Highest GG, the highest GG found in each prostate; GP3, GP4, GP5, Gleason Pattern 3, 4, 5 respectively; Age, age at recruitment; US vol, Ultrasound volume of prostate; PCa, prostate cancer; All areas measured from scans of H&E slides (see methods); for GP3 and GP4 amount calculation see results; GG2^{+5} indicates GG2 cancer with a tertiary GP5 (<5% of all PCa); PUR-1-4, Prostate urine risk signatures 1-4.

	**RPx data**							**Biopsy data**							**PUR signatures**			
	**GG**	**Majority GG, %GP4**	**Highest GG, %GP4**	**prostate area (mm^2)**	**Tumor area (mm2)**	**GP3 area**	**GP4 area**	**Age**	**PSA**	**GG**	**US vol**	**Total Biopsy Cores taken**	**PCa-Positive Cores**	**% cores PCa positive**	**PUR-1**	**PUR-2**	**PUR-3**	**PUR-4**
RPx 1	GG1	GG1, (+GP4<2%)	GG1, (+GP4<2%)	5269.9	486.4	481.6	4.8	54	5.5	GG1	54.5	10	3	30.0	0.45	0.31	0.20	0.04
RPx 2	GG2	GG2, <10% GP4	GG2, <10% GP4	3339.0	341.2	324.1	17.1	64	15	GG1	45.8	8	4	50.0	0.15	0.33	0.42	0.11
RPx 3	GG2	GG2, <10% GP4	GG2, <10% GP4	4347.6	531.8	505.2	26.6	52	5.8	GG1	68.0	12	2	16.7	0.10	0.30	0.46	0.14
RPx 4	GG2^{+5}	GG2, <10% GP4	GG5, GP4+5 ≥50% GP4	4606.0	254.5	233.9	20.5	70	8.4	GG3	59.0	10	3	30.0	0.01	0.08	0.48	0.44
RPx 5	GG2	GG2, 10-25% GP4	GG2, 10-25% GP4	3643.2	172.1	143.3	28.8	66	5.2	GG2	35.2	9	2	22.2	0.17	0.34	0.39	0.10
RPx 6	GG2	GG2, <10% GP4	GG2, 25-50% GP4	4529.7	364.0	302.0	62.0	68	6.7	GG2	36.1	28	18	66.7	0.00	0.03	0.35	0.62
RPx 7	GG3	GG2, 25-50% GP4	GG3, 50-75% GP4	11991.1	92.9	54.8	38.0	67	10.3	GG2	85.6	9	1	11.1	0.23	0.35	0.35	0.08
RPx 8	GG3	GG3, 50-75% GP4	GG3, 50-75% GP4	3729.6	158.7	60.3	98.4	65	7.4	GG2	46.0	23	3	13.0	0.01	0.08	0.47	0.44
RPx 9	GG3	GG3, 75-90% GP4	GG3, 75-90% GP4	3851.4	664.4	122.2	542.2	75	2.9	GG3^{+5}	38.0	10	8	80.0	0.00	0.04	0.40	0.56
